# Information and communication technologies: where are persons with intellectual disabilities?

**DOI:** 10.1186/s13584-018-0282-4

**Published:** 2019-01-09

**Authors:** Shirli Werner, Carmit-Noa Shpigelman

**Affiliations:** 1 Paul Baerwald School of Social Work and Social Welfare, Hebrew University of Jerusalem, Jerusalem, Israel; 2 Department of Community Mental Health, Faculty of Social Welfare and Health Sciences, University of Haifa, Haifa, Israel

**Keywords:** Accessibility, Information and communication technologies, Intellectual disabilities

## Abstract

Individuals with disabilities are entitled to equal access to information and communication technologies (ICT), including the Internet. The study to which this commentary refers has shown that over time (between 2003 and 2015), Internet access by persons with disabilities has increased, but a gap still exists between people with and without disabilities. One population that has been excluded from this study is that of individuals with intellectual disabilities. This is unfortunate because these individuals may face an even greater gap than others in access to the Internet. In this commentary we review the state of ICT use specifically by individuals with intellectual disabilities, and make a few recommendations for future ICT research and for reducing this gap.

The Convention on the Rights of Persons with Disabilities (CRPD) advocates the full and effective inclusion of persons with disabilities in all realms of life. Article 9 stresses that individuals have a right to participate fully in all aspects of life on an equal basis with others, with equal access to information and communication technologies (ICTs) and systems, including the Internet. In the Western world, ICTs, in particular social media, are a basic ingredient of interactions between individuals [1]. ICTs are of great importance for all individuals in society, including those with disabilities. ICTs provide a means of gaining information, accessing entertainment, and socializing [2].

To map some of the benefits of ICT use, the study conducted by Lissitsa and Madar [3] differentiated between using the Internet to gain human capital vs. social capital. Human capital relates to Internet use to seek information, including information related to health, products, and events [4]. Social capital refers to the ability to communicate with others using e-mail and other types of social media [3].

In their comprehensive study, based on data derived from the Annual Social Surveys of Israel’s Central Bureau of Statistics, Lissitsa and Madar [3] examined trends of Internet and digital use by persons with disabilities between 2003 and 2015. The researchers were able to obtain information on over 90,000 respondents, of whom over 22,000 reported having a disability. Looking at the half-full glass, they find that over time Internet access and digital uses by disabled persons continuously increased. The half-empty glass, however, shows that the gap between disabled and non-disabled persons persists, especially when disability is associated with other disadvantaged statuses.

This extensive study is of great importance, but one population was not represented because it was largely excluded from the Annual Social Survey: individuals with intellectual disabilities. These disabilities, identified before the age of 18 years, are characterized by significant limitations in both intellectual functioning and adaptive behavior. They affect conceptual, social, and practical adaptive skills [5].

The exclusion of this population from the current study is unfortunate because this particular population group is likely to experience a greater disadvantage in their access to ICTs than other disabled populations [6]. Cognitive and linguistic limitations of persons with intellectual disabilities are only part of the reason why this population is limited in their use of ICTs. Other important barriers include lack of appropriate training, lack of ongoing support, frequent changes in website interfaces, economic barriers, attitudinal barriers (a tendency to shelter these individuals from Internet use), and organizational culture [7].

Studies have repeatedly shown that persons with intellectual disabilities, similarly to other persons with a disability as well as members of society at large, can gain many benefits from the use of the Internet. In line with the human and social capital benefits suggested by Lissitsa and Madar [3], among the benefits of ICTs for persons with intellectual disabilities are greater social interactions, connectedness, participation in mutual support groups, and access to information [8, 9]. A recent review that focused on social media use by people with intellectual disabilities found that this online environment has a potential for positive social and emotional experiences for this population in the area of friendships, development of social identity and self-esteem, and enjoyment [10]. Additional benefits of ICT use by people with intellectual disabilities include increased opportunities for education, creativity, learning, communication, and civic engagement [11].

A shortcoming of previous research on ICT patterns and rates of use by persons with disabilities is its heavy focus on persons with physical disabilities or health conditions [12–14]. Much less research has focused on ICT patterns of use by persons with intellectual disabilities [15, 16]. The scant available research in this area has been based largely on relatively small samples of users, and it focused mainly on their subjective experiences [10, 17]. The lack of representation of persons with intellectual disabilities in ICT research, especially in national and international social and digital surveys, may be the result of the assumption that these online environments are not suitable for persons with intellectual disabilities because of their difficulty in understanding the associated risks, which include, for example, divulging confidential personal information to strangers and friends, and being exposed to online forms of fraud, bullying, and harassment [7, 18]. To overcome these barriers and to ensure that persons with intellectual disabilities enjoy the full potential and projected beneficial effects of online environments, training of safe use should be conducted to both the persons with intellectual disabilities and their family member and/or service provides who may provide them an ongoing support [16, 19].

We recommend that future ICT research apply an inclusive approach and target persons with intellectual disabilities. It is important to gain a better understanding of the patterns of use of this population, including the opportunities and access barriers of persons with intellectual disabilities, and compare their patterns of use with those of other population groups, with and without disabilities. More research is needed to assess the digital divide between subgroups of the disability community [10]. Researchers should survey persons with intellectual disabilities from various residential settings, including those who live in supported living arrangements. It is also important to measure ICT use not only with respect to computers, as reported in the Annual Social Surveys of Israel’s Central Bureau of Statistics [3], but also regarding other digital devices, such as tablets and smartphones, because of the massive growth in the use of these devices by the general population, including persons with disabilities. Future research should also include an assessment of factors that may promote or impede ICT use by persons with intellectual disabilities, such as their attitudes toward ICT use, given that attitudes were found to be associated with usage rate [20]. Note that the attitudes of persons with intellectual disabilities toward ICT use may be influenced by the attitudes and behaviors of individuals close to them, such as family members and professionals [19].

## Conclusions

The study conducted by Lissitsa and Madar [3] contributes to the understanding of how persons with disabilities use ICT. It also highlights the differences in ICT usage patterns among demographic subgroups within the disability community. However, ICT use should also be examined among persons with different types of disability, and especially persons with intellectual disabilities who often encounter access barriers.

In this sense, it is important to allow equal access to online applications, for example, by enforcing cognitive accessibility of websites. Using plain language has been found to be an effective strategy for giving persons with intellectual disabilities greater access to complex information [21]. It is also important to include technological skills in educational programs for persons with intellectual disabilities. Recently, national initiatives have been launched (http://nethaver.com, https://www.wai-not.org) to create accessible online social environments for persons with intellectual disabilities.

Technological developments in ICT use are of great importance, but at the same time they raise a question about whether society should promote the development of separate and accessible platforms for people with disabilities, or whether the existing platforms should be adapted to meet the needs of all citizens, including those with various types of disabilities.

## Abbreviations

CRPD: Convention on the Rights of Persons with Disabilities
ICT: Information and communication technologies
